# What evidence exists regarding the impact of biodiversity on human health and well-being? A systematic map protocol

**DOI:** 10.1186/s13750-024-00335-4

**Published:** 2024-04-27

**Authors:** Honghong Li, Raf E. V. Jansen, Charis Sijuwade, Biljana Macura, Matteo Giusti, Peter Søgaard Jørgensen

**Affiliations:** 1 Global Economic Dynamics and the Biosphere, Royal Swedish Academy of Sciences, Stockholm, Sweden; 2 Stockholm Resilience Centre, Stockholm University, Stockholm, Sweden; 3 Stockholm Environment Institute, Stockholm, Sweden; 4 School of Sustainability, Civil and Environmental Engineering, University of Surrey, Guildford, Surrey GU2 7XH UK

**Keywords:** Biodiversity loss, Ecosystem services, Planetary health, Evidence synthesis, Sustainability

## Abstract

**Background:**

Global biodiversity is rapidly declining, yet we still do not fully understand the relationships between biodiversity and human health and well-being. As debated, the loss of biodiversity or reduced contact with natural biodiversity may lead to more public health problems, such as an increase in chronic disease. There is a growing body of research that investigates how multiple forms of biodiversity are associated with an increasingly diverse set of human health and well-being outcomes across scales. This protocol describes the intended method to systematically mapping the evidence on the associations between biodiversity from microscopic to planetary scales and human health and well-being from individual to global scales.

**Methods:**

We will systematically map secondary studies on the topic by following the Collaborations for Environmental Evidence Guidelines and Standards for Evidence Synthesis in Environment Management. We developed the searching strings to target both well established and rarely studied forms of biodiversity and human health and well-being outcomes in the literature. A pairwise combination search of biodiversity and human health subtopics will be conducted in PubMed, Web of Science platform (across four databases) and Scopus with no time restrictions. To improve the screening efficiency in EPPI reviewer, supervised machine learning, such as a bespoke classification model, will be trained and applied at title and abstract screening stage. A consistency check between at least two independent reviewers will be conducted during screening (both title-abstract and full-text) and data extraction process. No critical appraisal will be undertaken in this map. We may use topic modelling (unsupervised machine learning) to cluster the topics as a basis for further statistical and narrative analysis.

**Supplementary Information:**

The online version contains supplementary material available at 10.1186/s13750-024-00335-4.

## Background

The biodiversity hypothesis states that loss of biodiversity (e.g. declining microbial diversity) or reduced contact with natural biodiversity leads to declines in human health and well-being (e.g. through immune dysfunction) [1, 2]. According to the World Health Organisation, human health is defined as “a state of complete physical, mental and social well-being and not merely the absence of disease or infirmity” [3]. Biodiversity is the variability among living organisms from the genetic to the ecosystems level” (https://www.cbd.int/convention/articles/?a=cbd-02), defined by Convention on Biological Diversity (CBD). The biodiversity hypothesis grew out of a set of hypotheses about the causes of increases in certain auto-immune/non-communicable diseases. In 1989, Strachan proposed the “Hygiene hypothesis” arguing that more unhygienic states, larger family sizes and overcrowding are associated with lower prevalences of certain non-communicable diseases [4]. This hypothesis was heavily criticised, and multiple studies later found that childhood infections did not offer complete protection against the emergence of allergy-related diseases [5–7]. To account for these discrepancies, the “old friend hypothesis”, proposed by Rook in 2003, argues that due to continuous exposure to different microorganisms through untreated water and food, humans coevolved with these organisms and developed an immunotolerance towards them [8, 9]. Thus, defective immunoregulation caused by limited interaction with nature increases human’s chronic inflammatory disorders and allergy-related diseases [9]. However, so far, the interactions with nature have mainly been studied for a limited set of health outcomes and biodiversity forms (e.g. microbiota and green space).

**Fig. 1:**
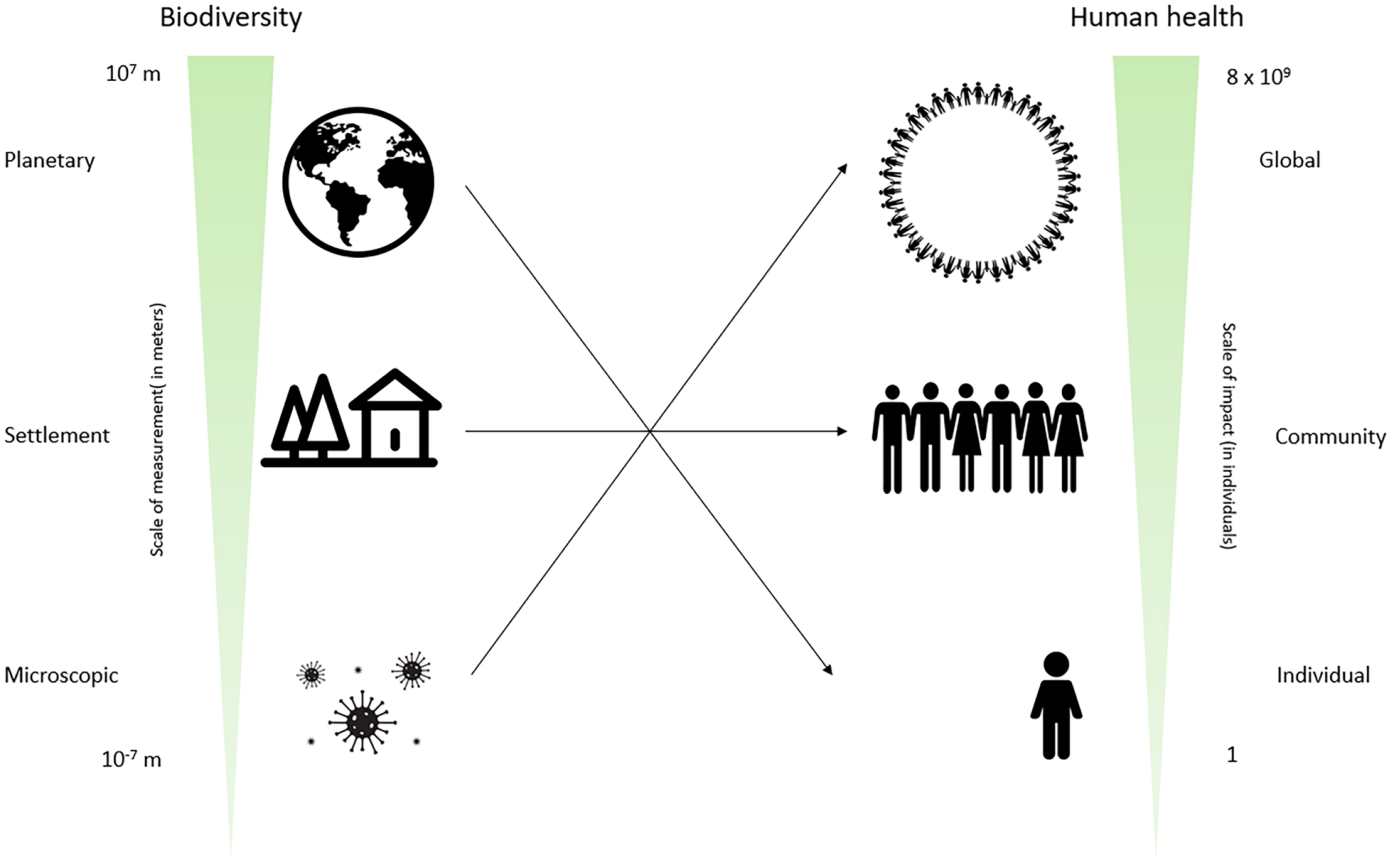
Schematic figure of the relationships between biodiversity and human health and well-being across different scales

Human health is rooted in biodiversity and has been considered as the ultimate ecosystem services—the conditions and processes within natural ecosystem that can sustain and fulfil human life [10, 11]. The megatrend of biodiversity loss is likely undermining the contributions of nature to human health and well-being [12]. An increasing set of human health problems have been widely linked to the decrease of environmental biodiversity [13] or lack of interactions with nature [14–22]. It has been recognised that (1) associations between nature exposure and human health vary when different forms/components of biodiversity and human health are measured; (2) the impacts of biodiversity on health vary when different measurements of biodiversity are assessed, e.g. actual versus perceived species richness; (3) most health outcomes (as high as 80%) are self-reported and not clinically assessed, which presents a challenge as self-reported outcomes are typically not clearly or consistently defined throughout the literature [23, 24]. Despite these trends, the literature on biodiversity and health outcomes is surprisingly scattered. This is because the associations between biodiversity and human health and well-being requires many different approaches depending on the components of health and biodiversity of interested. Further, the relationships are likely multifaceted and could vary across spatial and temporal scales and forms of life and outcomes of health (Fig. 1). These heterogeneous and scattered findings call into question if the biodiversity hypothesis remains significant when applied to different forms of biodiversity and human health outcomes, and yet, there is no overarching summary of this evidence.

In this protocol we outline the methods underlying a study that aims to summarize the evidence for a general biodiversity hypothesis of human health. Doing so requires a multipronged approach to the relationships between biodiversity and human health and well-being across different scales. Systematic maps were created in the social science research space to provide a framework for researchers to address broader and more exploratory research topics. Therefore, we aim to conduct a systematic map to incorporate heterogeneous studies from different research fields, describe the literature-based evidence, and identify knowledge gaps and clusters. With this systematic map, researchers and policy makers will be supported with a holistic view on prioritizing sustainable biodiversity conservation practices to secure human health and well-being at different scale.

### Stakeholder engagement

Referring to previously mentioned definitions for stakeholders [25], we define stakeholders or rights holders as any individual, group or representative of an organisation that is affected by or can affect an issue or a decision. We initially identify individual stakeholders with our team members’ known contacts and then expand the stakeholder selection through snowballing. At an early stage, to set the scope of the map, prioritise research questions, develop searching strategy, and share and endorse the map, we contact individual stakeholders through email. Using the interest and influence matrix analysis [26], we will mainly contact the organisational representatives at Intergovernmental Science-Policy Platform on Biodiversity and Ecosystem services (IPBES), Convention on Biological Diversity (CBD), World Health Organization (WHO), Food and Agriculture Organization of the United Nations (FAO), and World Wildlife Fund (WWF). When necessary, we will organise meetings with individuals or groups of stakeholders. All stakeholders we engaged were listed in Additional file 1.

### Objectives of this systematic map

This systematic map primarily aims to map out the existing evidence of biodiversity impacts on human health and well-being across scales.

### Main research question

What forms of biodiversity affect which components of human health and well-being, at what scales?

- What combinations of biodiversity and health have been assessed with what volume of evidence?
- What spatial scales has the evidence been assessed at?
- How do methods of inquiry vary with combinations of biodiversity and health?

### Secondary research questions

- What is the distribution of the evidence by year and publication types (e.g. narrative review, systematic map, systematic review, meta-analysis and primary studies)?
- What are the current research gaps based on secondary studies?

### Definitions of the question components

**Population**: Human(s) regardless of location, population group, age, or gender.

**Exposure(s)**: Any form of biodiversity in any type of ecosystems: (1) blue space, (2) food production, (3) green space, (4) microbiome, (5) protected areas, and (6) general biodiversity (to capture emergent subcategories that have not been defined or covered by the above groups). The definitions of biodiversity forms were listed in Additional file 2.

**Comparator(s)**: Contact with one or more biodiversity forms in any type of ecosystem, where either difference in biodiversity level or exposure level is well-known or measured. For narrative reviews, systematic maps, or systematic reviews, comparator(s) may either be implicit or none.

**Outcome(s)**: The relationships of biodiversity with any component of human health and well-being in the following categories: (1) atopic and respiratory disease, (2) cancer-related disease, (3) cardiovascular and respiratory disease, (4) food and nutrition security, (5) objective well-being, (6) subjective well-being (including mental health), (7) infectious disease, (8) other non-communicable diseases, and (9) general human health (to capture emergent subcategories of human health that have not been defined or covered by the above groups). The definitions of health components were listed in Additional file 2.

## Methods

We started this protocol by following the CEE guidelines for a systematic map—“Guidelines and Standards for Evidence Synthesis in Environmental Management (version 5.1, 2022) [27]. The RepOrting standards for systematic Evidence synthesis (ROSES) [28] is used as a checklist for this systematic map protocol (Additional file 6).

### Scoping search and benchmark papers

The scoping search exercise in Web of Science across all databases revealed that having one search string for each concept of biodiversity (e.g., biodivers* OR “biological divers*” OR “species richness” OR “ecosystem divers*”) and human health (e.g., “human health” OR “human health status” OR “human physical health” OR “human mental health”) returned a large number of results. By analysing the searched results with publication years and Medical Subject Headings (MeSH) in Web of Science, we observed an expanding scope of research in both biodiversity and human health and well-being. To capture different forms of biodiversity and ecosystems that might not be captured by general biodiversity string, we developed five additional search strings for biodiversity subtopics by referring to the well-developed and rarely mentioned biodiversity forms from literatures. Similarly, to capture different components of human health and well-being that the general health search string might not capture, we developed eight additional search strings for human health and well-being subtopics, which aims to reflect the major non-communicable and communicable diseases. We used narrow definitions for each subtopic (e.g. green space is mainly defined with all urban land covered by vegetation of any kind) to avoid overlapping searching strings between the subtopics (e.g., between green space and food production) and reduce the irrelevant search results from the pairwise combinations (Additional file 2).

We developed the initial search strings by checking the search terms used in the relevant review articles and referring to the author keywords listed in the benchmark papers. When necessary, the other relevant terms were obtained from title and abstract by text-mining with the “litsearchr” package in RStudio [29]. To improve the sensitivity of search strings, truncated search terms and wild cards (e.g., *, ?, $) were used to cover the alternative forms of the search terms. The synonyms of the terms were also checked. On the other hand, quotation marks (“”) and brackets ({}) were used to improve the specificity by limiting the search results into exact terms. The search strings were mainly tested in Web of Science, and results were restricted to documents written in English.

We define benchmark papers as articles that could accurately represent our interests and serve as a standard for other papers. When selecting a benchmark paper, the following criteria were checked: (1) studies must be conducted on humans (2) studies must contain the contact with biodiversity and the health outcomes and (3) the relationships must be clearly stated, with the direction being that of biodiversity impacts on human health and well-being. We identified a short list of benchmark papers by narrowing down the scoping search into the studies we were most interested in and selected the studies that met all the three criteria.

The developed strings will be piloted to ensure the benchmark paper(s) for each pairwise combination of subtopics is captured. By doing this, the search strings are validated. If the strings fail to capture the benchmark papers, we will either modify the strings or explain why the benchmark papers were not captured. All pairwise search strings were checked against benchmark papers before conducting the real searching.

#### Searching in databases

The literature search will be conducted with the databases available through Stockholm University Library and following the systematic searching rules for environmental evidence [30]. We will search PubMed provided by the National Library of Medicine, Scopus, and all databases included in the Web of Science platform (Web of Science Core Collection [1986-present], KCI-Korean Journal Database [1980-present], MEDLINE [2002-present], and SciELO Citation Index [2002-present]). In Web of Science, the search strings will be entered into the fields of article title, abstract and author keywords in advanced search mode with “exact search” turned on. In Scopus and PubMed, the same search strings were searched in article title, abstract and author keywords as in Web of Science with some adaptations to the databases’ own search rules.

Due to the expansive scope of biodiversity and human health research landscape, searches will initially focus on evidence synthesis outputs. Thus, searching will be refined into secondary studies. The review of reviews will help us understand the spread of the literature, where evidence syntheses are distributed, and identify the evidence gaps. Therefore, a study design clause (e.g. “narrative review”, “systematic review”, “systematic map”, and “meta-analysis”) will be added to refine the results into secondary studies. Thus, the search will be conducted systematically by running biodiversity search, human health search and study design search independently and then combining the searched sets with Boolean operators AND within each combination of biodiversity and human health topics.

To target the relevant study populations, an exclude clause is used to exclude the studies not conducted on human subjects with Boolean operators NOT or AND NOT. Finally, an English language filter will be applied without any restrictions on publication date. To keep track of the publications that may have been retracted, the search results will be exported and managed in Zotero if necessary.

When the availability or representativity of secondary studies is low, we may also search for primary studies. Thus, if time allows, we will modify the search strings to search for primary studies. The final developed search strains and benchmark paper list are presented in Additional file 3 and Additional file 4, respectively. To enable the review and re-use of our search strings, we will submit the final searching strings to the Centre for Agriculture and Bioscience International (CABI) Digital Library searchRxiv https://www.cabidigitallibrary.org/journal/searchrxiv

### Screening process

The results from the Web of Science platform across all databases, Scopus and PubMed will be imported into EPPI-Reviewer (a web-based software program for managing and analysing data in literature reviews) [31]. Duplicates within each database, between databases and between 54 pairwise groups will be identified with the similarity score calculated by EPPI-Reviewer (0–1, with 1 representing identical citation information of the grouped literatures and 0 indicating that the citation information is completely different between the grouped literatures). To manage the duplicates, we will (if necessary) gradually decrease the threshold of similarity score from 0.86 to 0.80 and automatically remove the duplicates. After this, we will manually remove other plausible duplicates with similarity scores lower than the score used for automatic deduplication based on the comparison of citation information (e.g. article title, author, publication date).

An article will be first screened based on its title and abstract, and if it is marked as relevant, the full-text screening will be conducted at the next stage. The dataset subsampled from all pairwise groups (2.5–5% from each group) will be deduplicated and screened independently by two reviewers during the screening process at both title and abstract and full-text screening stages. To validate the inclusion/exclusion criteria and minimise bias during screening, a pre-screening with consistency check prior to the formal screening process will be conducted by two independent reviewers on a random subset (up to 5%) of the literatures. At the title and abstract screening stage and whenever the reviewer is not quite sure whether to include or exclude the article, the reviewer could mark it as “include for second opinion”. When the abstract is missing, the reviewer is asked to check the abstract through the article link provided within EPPI and then make his/her decision. During the screening process, reviewer(s) are asked to mark the main reason why they exclude the article. Cohen’s kappa tests will be used to check the consistency of independent screening [32]. A kappa score of 1 represents complete agreement, whereas a score of 0 represents a random agreement between the reviewers. A kappa score of > 0.6 indicates substantial agreement, while the score of ≤ 0.6 indicates insufficient agreement between the reviewers. Whether the score is > 0.6 or ≤ 0.6, we will first discuss and reconcile the disagreements. Disagreements between the reviewers will be reconciled and, if necessary, discussed with a third reviewer. If the score is ≤ 0.6, we will clarify the eligibility criteria and repeat the consistency check until a consistency score of > 0.6 is achieved.

To improve the screening efficiency based on title and abstract, we will build a classification model or classifier with supervised machine learning support. The model is built on a computer algorithm that can be trained by human reviewer (s) with the included/excluded studies. Thus, the machine can learn and predict the relevance of unscreened literature. The screened articles from consistency check (composed of records screened at random mode in EPPI reviewer by at least two reviewers) is used as part of the training dataset to build up the model. As part of this process, we may also train the priority screening system to speed up the building of the model or as a backup in case of poor performance of the classification model. Under priority screening mode, the machine will learn from the training dataset and push the most relevant literature to the human reviewer first.

To make sure that the machine was trained with sufficient included/excluded examples, the minimum number of included papers was set at 20 before switching into priority mode from the random mode. Depending on the distinction between includes and excludes from the title and abstract, we will manually screen certain number of the literature until the performance of the binary classifier is more reliable. The performance of the binary classifier will be validated by checking the parameters of the model such as accuracy, precision, and recall. If the classifier works well, we will apply the model to the remaining unscreened literatures. Thus, the remaining literatures will be organised into 10 categories with scores representing the likelihood of relevance (range from 0 to 99, with 99 indicating highly relevant and 0 indicating not relevant at all). Based on the validation of the classifier model (accuracy, AUC, precision, and recall), we will decide a cut-off point when the relevance score is acceptable for inclusion or exclusion. After this, we may use priority screening to check the literatures within an ambiguous range of the score e.g., 20–80), where the model is uncertain whether the literature should be included or excluded.

During the full-text screening, we will extract the metadata in EPPI reviewer according to the coding system (Additional file 5). Alternatively, if the volume of literature after screening based on title and abstract is very large, we may use a unsupervised or semi-supervised machine learning such as topic modelling rather than full-text screening to cluster the topics and synthesise the evidence narratively. Without any human training, topic modelling can find the thematic topics with the computer algorithm that can automatically identify the clusters of words from the included literature. The clusters of words occurring together are used to describe a topic and the topic score of each document is calculated based on the associations between the words in the document and the specific topic. Based on the balance between adequate detail and interpretability of the topics, we will decide the number of topics in the final model.

### Eligibility criteria

As we will focus mainly on secondary studies, we predefined the criteria by checking the population, exposure, comparator, and outcomes (PECO) of the most relevant studies. To be considered eligible for inclusion, studies must meet the requirements described in the following subsections.

#### Population

Studies mention humans and focus on the health and well-being of human population at an individual, household, community, country, continental region, or global population level.

#### Exposure

Studies mention exposure to one or more forms of biodiversity (e.g. green space, blue space, microbiome, food protection, protected area and general biodiversity) and address biodiversity impacts on human health and well-being.

#### Comparators

Comparison should be made between either biodiversity levels or exposure levels. Biodiversity metrics in the studies should be able to reflect the diversity of nature and the variety of organisms.

When exposed to ecosystems with different levels of biodiversity, the biodiversity level needs to be either measured or well known. For example, the actual and/or perceived biodiversity (such as species richness, abundance, and Shannon diversity) should be reported, and the comparator(s) could be high diversity vs. low diversity.

When exposed to the same biodiversity level, the exposure level should be measured or well known. For example, the actual and/or proxy measures of interactions or contacts with the same ecosystems by participants should be reported. This may include the actual measure of time spent in the ecosystems, frequency of visits to the ecosystem or a proxy measure of cumulative opportunity (e.g., normalised difference vegetation index [NDVI], satellite-derived vegetation indices, percentages of green and blue areas) and proximity (e.g. distance to the nearest green or blue space, number of parks near home). Thus, the comparator (s) could be no contact vs. contact, long contact time vs short contact time, high-frequency contact vs low-frequency contact, and high proximity vs low proximity to biodiversity.

#### Outcomes

Studies must report the relationships of any type of biodiversity form with any human health and well-being component. The outcomes could be either observable (e.g., prevalence of disease within a population, morbidity, mortality) or self-reported (e.g., mental health, well-being). Ideally, the measured outcomes should reflect the degree of health or well-being observed in participants.

#### Study design

Peer-reviewed secondary studies such as systematic reviews, narrative reviews, and meta-analyses will be included. Protocols, commentaries, editorials, personal observations, theoretical works, and opinion pieces where data are not systematically assessed will be excluded. Perspectives that present new summarises and grey literature that synthesised evidence on relationships between human health and well-being, such as organisational reports from CBD, IPBES, WHO or FAO, might be included. We may include peer-reviewed primary studies when the representativeness and availability of secondary studies are low.

#### Language

English.

#### Publication time

No restrictions.

### Data coding strategy

In line with our research questions and inclusion/exclusion criteria, we have defined and developed a comprehensive data coding framework (Additional file 5). However, it is important to note that the purpose of this systematic map is not to synthesise study findings. We will therefore explore the types and the spread of evidence in the literature rather than extracting the results from each study. Thus, we will focus on the method part of each study when extracting information during full-text screening. If the relevant information is not provided in these sections or some are missing, we may review the introduction section. If the study does not have a clear structure (no subtitles for abstract or method), we may search throughout the whole text. When important information is not accessible or unclear, we will mark the associated fields with the term “not available”. We will not extract the information from individual studies synthesized in secondary studies.

This study aims to map out the existing evidence from secondary studies. Thus, metadata extraction will focus on the more general variables related to the synthesis from secondary studies. After mapping out the evidence gaps with evidence synthesis studies, we may dive into the primary studies to fill in the evidence gaps, which will be coded in more detail and with different codes.

We will register the coding framework in the data extraction section of EPPI-Reviewer and code each included study during full-text screening. The following necessary information will be coded during the process: (1) general information (e.g., study ID, study design, person screening, date screened); (2) bibliographic information of each study (author [s], title, abstract, year of publication, journal, study site); (3) PECO of each study. If the study does not specify the forms of biodiversity and/or human health components, we will analyse their searching strings (if provided). The study design of secondary studies will be coded as described by the authors. The consistency of data extraction will be checked by at least two reviewers, with disagreements resolved through discussion or in consultation with a third reviewer if necessary.

### Study mapping and presentation

Based on the metadata we collect with the coded database detailing the specifics of the variables from each included literature or alternatively with the outcomes from topic modelling, we will explore the general trends of the evidence distribution by factors such as by year, study design, geographical location, combinations of biodiversity and human health and well-being. When necessary, we may use Evi-Atlas [33] to visualize the geographical distribution of the evidence.

We will use evidence heat maps to present the clusters of evidence and identify the evidence gaps for the combinations of biodiversity and human health. For example, we will draw a map by cross tabulating the exposure types in terms of biodiversity forms as one variable and the outcomes in terms of human health and well-being components as another variable, then present the volume of evidence within each cell with the number of studies (Table 1). We may also consider using EPPI-Mapper or the available R packages to build an interactive map.

**Table 1:**
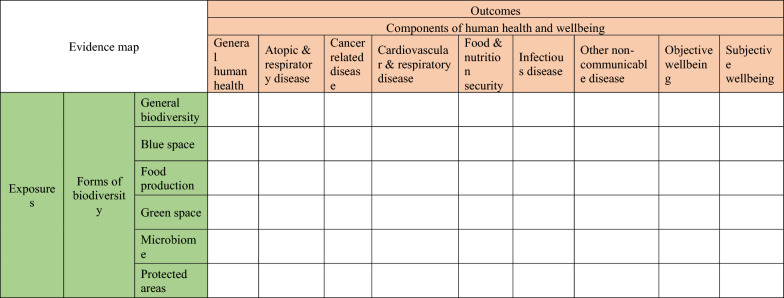
Evidence map of the relationship between biodiversity and human health and wellbeing

The narrative synthesis of this map will follow a framework that highlights the relationship between biodiversity and human health and well-being across different scales. Furthermore, we may use the identified gaps from this map as a starting point for a systematic review or meta-analysis, which will help us to populate the gaps with primary studies. When necessary, the theory of change [34] will be used to interpret the findings and categorise them based on similarities and differences.

## Supplementary Information

**Additional file 1: **List of stakeholders that are engaged or will be engaged in this systematic map.

**Additional file 2: **Definitions for the forms of biodiversity and the components of human health and wellbeing.

**Additional file 3: **Searching strings developed for Web of Science, Scopus and PubMed.

**Additional file 4: **Benchmark paper list.

**Additional file 5: **Data coding system.

**Additional file 6.** ROSES for Systematic Map Protocols. Version 1.0.

## Data Availability

Not applicable.
