# Correction: What evidence exists regarding the impact of biodiversity on human health and well-being? A systematic map protocol

**DOI:** 10.1186/s13750-024-00338-1

**Published:** 2024-05-20

**Authors:** Honghong Li, Raf E. V. Jansen, Charis Sijuwade, Biljana Macura, Matteo Giusti, Peter Søgaard Jørgensen

**Affiliations:** 1 Global Economic Dynamics and the Biosphere, Royal Swedish Academy of Sciences, Stockholm, Sweden; 2 Stockholm Resilience Centre, Stockholm University, Stockholm, Sweden; 3 Stockholm Environment Institute, Stockholm, Sweden; 4 School of Sustainability, Civil and Environmental Engineering, University of Surrey, Guildford, Surrey, GU2 7XH UK

**Correction: Environmental Evidence (2024) 13: 11**

10.1186/s13750-024-00335-4

In this article the funding from ‘[***P.S.J. acknolwedges funding from the IKEA foundation, Marianne and Marcus Wallenberg Foundation, the Marcus and Amalia Wallenberg Foundation, the Erling-Persson Foundation, the European Union (ERC, INFLUX, 101039376), and the Swedish Research Council Formas (2020****–00371)*’ was omitted in the acknowledgement section.

The complete acknowledgement is given below,

We acknowledge the Erling-Persson Foundation for supporting this work. We thank the stakeholders for providing comments and suggestions on developing search strings. We wish to thank Neal Haddaway for his help on the consultation of a systematic map and Melissa Barton for reviewing the manuscript and providing comments. *P.S.J. acknolwedges funding from the IKEA foundation, Marianne and Marcus Wallenberg Foundation, the Marcus and Amalia Wallenberg Foundation, the Erling-Persson Foundation, the European Union (ERC, INFLUX, 101,039,376), and the Swedish Research Council Formas (2020 − 00371).*

The Original article has been corrected.

