# Supplementary material for: What evidence exists regarding the impact of biodiversity on human health and well-being? A systematic map protocol
Source: Environ Evid. 2024 Apr 27;13:11. doi: 10.1186/s13750-024-00335-4 (PMC11378774; doi:10.1186/s13750-024-00335-4)
Supplement: Supplementary file 1 — Additional file 1: List of stakeholders that are engaged or will be engaged in this systematic map. [file 13750_2024_335_MOESM1_ESM.docx]

**Supplementary Table 1**: List of stakeholders that are engaged or will be engaged in this systematic map.

| **Name** | **Organization/Institute** | **Location/Country** | **Contact** | **Co-authors** |
| --- | --- | --- | --- | --- |
| Miina Porkka | Aalto University | Helsinki/Finland | [minna.porkka@aalto.fi](mailto:minna.porkka@aalto.fi) | No |
| Max Troell | Royal Swedish Academy of Sciences | Stockholm/Sweden | [max@beijer.kva.se](mailto:max@beijer.kva.se) | No |
| Garry Peterson | Stockholm University | Stockholm/Sweden | [garry.peterson@su.se](mailto:garry.peterson@su.se) | No |
| Biljana Macura | Stockholm Environment Institute | Stockholm/Sweden | [biljana.macura@sei.org](mailto:biljana.macura@sei.org) | Yes |
| Matteo Giusti | University of Surrey | Surrey/UK | [m.giusti@surrey.ac.uk](mailto:m.giusti@surrey.ac.uk) | Yes |
|  |  |  |  |  |
|  |  |  |  |  |
| XXX | IPBES |  |  |  |
| XXX | WWF |  |  |  |
| XXX | CBD |  |  |  |
| XXX | WHO |  |  |  |
| XXX | FAO |  |  |  |
| Anonymous | Anonymous | Anonymous | Anonymous | Anonymous |
