# Supplementary material for: What evidence exists regarding the impact of biodiversity on human health and well-being? A systematic map protocol
Source: Environ Evid. 2024 Apr 27;13:11. doi: 10.1186/s13750-024-00335-4 (PMC11378774; doi:10.1186/s13750-024-00335-4)
Supplement: Supplementary file 2 — Additional file 2: Definitions for the forms of biodiversity and the components of human health and wellbeing. [file 13750_2024_335_MOESM2_ESM.docx]

**Supplementary file 2** Definitions for the forms of biodiversity and the components of human health and wellbeing

**1 Biodiversity** is defined as “the variability among living organisms from all sources, including, inter alia, terrestrial, marine and other aquatic ecosystems and the ecological complexes of which they are part; this includes diversity within species, between species and of ecosystems” (CBD).

- 1. **Blue space(s)**

Blue space in urban planning and design comprises all the areas dominated by surface water bodies or watercourses. It comprises multiple typologies, spanning natural, seminatural and manmade, in the form of rivers, coasts, lakes, canals, ponds, and sustainable drainage features.

**1.2 Food production**

Food production refers to the processes of growing, harvesting, and processing the food.

**1.3 Green space**

Urban green space is defined as all urban land covered by vegetation of any kind (WHO). This study mainly focuses on the urban green space, such as nature located close to urban areas, fit for recreational activities.

**1.4 Microbiome**

Microbiome is the community of microorganisms (such as fungi, bacteria, archaea, and viruses) that can usually live together in any given habitat (Wikipedia). Microbiota consists of all living members forming the microbiome.

**1.5 Protected area**

Protected areas or biosphere-conserved areas are locations which receive protection because of their recognised natural, ecological, or cultural values. Protected areas are areas where human presence, or the exploitation of natural resources is limited (Wikipedia).

**1.6 General biodiversity:**

General biodiversity is used to capture emergent subcategories not defined or covered by the above groups.

**2 Human health and wellbeing**

Human health and well-being are defined as “a state of complete physical, mental and social well-being and not merely the absence of disease or infirmity” (WHO).

**2.1 Atopic and respiratory disease**

Atopy is the tendency to produce an exaggerated immunoglobulin E (IgE) immune response to otherwise harmless substances in the environment. Allergic diseases (such as allergic asthma) are clinical manifestations of such inappropriate, atopic responses (Wikipedia).

**2.2 Cancer-related disease**

Cancer is a group of diseases involving abnormal cell growth with the potential to invade or spread to other parts of the body (Wikipedia).

**2.3 Cardiovascular and respiratory diseases**

Cardiovascular disease involves the heart or blood vessels (Wikipedia). Cardiovascular diseases are a group of disorders of the heart and blood vessels (WHO).

**2.4 Food and nutrition security**

Food security is a multidimensional concept characterised by four pillars: availability, access, utilisation, and stability (FAO, 1996). Nutrition is embedded in FAO’s definition of food security: that all people, at all times, have sufficient, safe and nutritious food for an active and healthy life. In its strategic framework 2010-2019, FAO’s first goal is reducing the number of hungry and ensuring adequate nutrition for all. <https://www.fao.org/3/me785e/me785e.pdf>

**2.5 Infectious diseases**

Infectious diseases are caused by pathogenic microorganisms, such as bacteria, viruses, parasites or fungi. The diseases can be spread, directly or indirectly, from one person to another.

**2.6 Subjective wellbeing (including mental health)**

**Subjective well-being** (perceived well-being collected by self-reports) includes psychological factors, such as our perceptions, confidence, fulfilment, sense of belonging and purpose. Subjective well-being varies between individuals. The World Health Organisation (WHO) states that mental health is a state of well-being in which an individual realises his or her own abilities, can cope with the normal stresses of life, can work productively and is able to contribute to his or her community.

**2.7 Other non-communicable disease**

Non-communicable disease is a disease that is not transmissible directly from one person to another.

**2.8 Objective wellbeing**

Objective well-being encompasses quality of life and the ability of people and societies to contribute to the world with a sense of meaning and purpose. Well-being is an overall measurement or evaluation of life. Objective well-being includes physical factors that widely account for our basic needs, such as food/nutrition needs, money, employment, housing, safety, physical and mental health, and human rights. Six major objective well-being dimensions include health, job opportunities, socioeconomic development, environment, safety, and politics ([Measuring objective and subjective well-being: dimensions and data sources | SpringerLink](https://link.springer.com/article/10.1007/s41060-020-00224-2))

**2.9 General human health**

General human health is used to capture emergent subcategories not defined or covered by the above groups.
