# Supplementary material for: What evidence exists regarding the impact of biodiversity on human health and well-being? A systematic map protocol
Source: Environ Evid. 2024 Apr 27;13:11. doi: 10.1186/s13750-024-00335-4 (PMC11378774; doi:10.1186/s13750-024-00335-4)
Supplement: Supplementary file 3 — Additional file 3: Searching strings developed for Web of Science, Scopus and PubMed. [file 13750_2024_335_MOESM3_ESM.docx]

**Supplementary file 3: Searching strings developed for Web of Science, Scopus and PubMed**

**Biodiversity strings:**

**1) General biodiversity**

**Web of Science**“biodivers*” OR “nature divers*” OR “biological divers*” OR “ecosystem$” OR “natural environment$” OR “species diversity” OR “species richness” OR “wild environment$” OR “wild area$” OR “ecological connectivity” OR “ecological resilience” OR “ecosystem health$” OR “environmental biodiversity” OR “functional trait$ diversity” OR “functional diversity” OR “ecosystem diversity” OR “landscape structure” OR “landscape diversity”

**Scopus**

“biodivers*” OR “nature divers*” OR “biological divers*” OR “ecosystem” OR “natural environment” OR {species diversity} OR {species richness} OR “wild environment” OR “wild area” OR {ecological connectivity} OR {ecological resilience} OR “ecosystem health” OR {environmental biodiversity} OR “functional trait diversity” OR {functional diversity} OR {ecosystem diversity} OR {landscape structure} OR {landscape diversity}

**PubMed**

“biodivers*” OR “nature divers*” OR “biological divers*” OR “ecosystem*” OR “natural environment*” OR “species diversity” OR “species richness” OR “wild environment*” OR “wild area*” OR “ecological connectivity” OR “ecological resilience” OR “ecosystem health*” OR “environmental biodiversity” OR “functional trait diversity” OR “functional traits diversity” OR “functional diversity” OR “ecosystem diversity” OR “landscape structure” OR “landscape diversity”

**2) Blue space
 Web of Science**

"blue space$” OR “bluespace$” OR “river$” OR “lake$” OR “pond$” OR “coast*” OR “beach*” OR "seaside" OR “waterfall" OR “inland water$” OR “lagoon” OR “riverine” OR “riparian” OR "dead sea" OR ((“stream” OR "canal$" OR "spring") AND "water")

**Scopus**

“blue space” OR “bluespace” OR “river” OR “lake” OR “pond” OR “coast*” OR “beach*” OR {seaside} OR {waterfall} OR “inland water” OR {lagoon} OR {riverine} OR {riparian} OR {dead sea} OR (({stream} OR “canal” OR {spring}) AND {water})

**PubMed**

"blue space*” OR “bluespace*” OR “river*” OR “lake*” OR “pond*” OR “coast*” OR “beach*” OR "seaside" OR “waterfall" OR “inland water*” OR “lagoon” OR “riverine” OR “riparian” OR "dead sea" OR ((“stream” OR "canal*" OR "spring") AND "water")

**3) Food production**

**Web of Science**

("biodivers*" OR “divers*” OR “crop divers*”) AND (“food product*” OR "agriculture" OR “livestock$” OR “fisheries” OR “aquatic culture” OR “aquaculture$” OR "harvest" OR “cropping system$” OR “agricultural production” OR “land-use” OR “agroecology” OR “agroforestry” OR “urban garden*” OR “home garden*”) OR “intercropping” OR “farm*” OR “agrobiodiversity”

**Scopus**

(“biodivers*” OR “divers*” OR “crop divers*”) AND (“food product*” OR {agriculture} OR “livestock” OR {fisheries} OR {aquatic culture} OR “aquaculture” OR {harvest} OR “cropping system” OR {agricultural production} OR “land-use” OR {agroecology} OR {agroforestry} OR “urban garden*” OR “home garden*”) OR “intercropping” OR “farm*” OR {agrobiodiversity}

**PubMed**

(“biodivers*” OR “divers*” OR “crop divers*”) AND (“food product*” OR “agriculture” OR “livestock*” OR “fisheries” OR “aquatic culture” OR “aquaculture*” OR “harvest” OR “cropping system*” OR “agricultural production” OR “land-use” OR “agroecology” OR “agroforestry”) OR “urban garden*” OR “home garden*”) OR “intercropping” OR “farm*” OR “agrobiodiversity”

**4) Green space**

**Web of Science**“green space$” OR “greenspace$” OR ("forest$" NOT ("plot$" OR "analysis" OR "algorithm*" OR "classifier")) OR "garden$" OR "gardener$" OR "gardening" OR “urban park$” OR "city park$" OR “greenness” OR “green area$” OR “green infrastructure$” OR “woodland$” OR "green* exposure” OR “green space exposure” OR “residential green*” OR “urban green” OR “vegetation” OR “greener$” OR “normalised difference vegetation index” OR “NDVI” OR “horticultural therapy” OR “forest therapy” OR “street tree$” OR “green roof$”

**Scopus**

“green space” OR “greenspace” OR (“forest” AND NOT (“plot” OR {analysis} OR “algorithm*” OR {classifier})) OR “garden” OR “gardener” OR {gardening} OR “urban park” OR “city park” OR {greenness} OR “green area” OR “green infrastructure” OR “woodland” OR “green* exposure” OR {green space exposure} OR “residential green*” OR {urban green} OR {vegetation} OR “greener” OR {normalised difference vegetation index} OR {NDVI} OR {horticultural therapy} OR {forest therapy} OR “street tree” OR “green roof”

**PubMed**

“green space*” OR “greenspace*” OR ("forest*" NOT ("plot*" OR "analysis" OR "algorithm*" OR "classifier")) OR "garden*" OR "gardener*" OR "gardening" OR “urban park*” OR "city park*" OR “greenness” OR “green area*” OR “green infrastructure*” OR “woodland*” OR "green exposure” OR “green space exposure” OR “residential green*” OR “urban green” OR “vegetation” OR “greener*” OR “normalised difference vegetation index” OR “NDVI” OR “horticultural therapy” OR “forest therapy” OR “street tree*” OR “green roof*”

**5) Microbiome**

**Web of Science**(“microbiome” OR “microbiota” OR “gut microbiota” OR “mycobiome” OR “virome” OR “microflora” OR “commensal bacteria*” OR “microbial” OR “oral microbiota” OR “bacterial” OR “fungal” OR “archaeal”) AND ("divers*" OR "biodivers*" OR “composition” OR “communit*” OR “structure” OR “dysbiosis” OR “community structure” OR “community composition”)

**Scopus**

({microbiome} OR {microbiota} OR {gut microbiota} OR {mycobiome} OR {virome} OR {microflora} OR “commensal bacteria*” OR {microbial} OR {oral microbiota} OR {bacterial} OR {fungal} OR {archaeal}) AND (“divers*” OR “biodivers*” OR {composition} OR “communit*” OR {structure} OR {dysbiosis} OR {community structure} OR {community composition})

**PubMed**

(“microbiome” OR “microbiota” OR “gut microbiota” OR “mycobiome” OR “virome” OR “microflora” OR “commensal bacteria*” OR “microbial” OR “oral microbiota” OR “bacterial” OR “fungal” OR “archaeal”) AND ("divers*" OR "biodivers*" OR “composition” OR “communit*” OR “structure” OR “dysbiosis” OR “community structure” OR “community composition”)

**6) Protected areas**

**Web of Science**

“Protected area$” OR “nature reserve$” OR “nature preserve$” OR “nature conserve$” OR “biosphere reserve$” OR “bioreserve$” OR “national park$” OR “wilderness area$” OR “community conserved area$” OR “biodiversity conservation” OR “marine reserve$” OR “marine conservation area$” OR “marine sanctuar*” OR “marine managed area$” OR “wildlife conservation” OR “marine conserved area$” OR “marine conserved territor*” OR “ocean conservation area$” OR “natural resource$ management” OR “community-based conservation$” OR “managed forest$” OR (“natural” AND (“monument” OR “feature”)) OR (“protected” AND (“landscape$” OR “seascape$”)) OR ((“habitat$” OR “species”) AND “management area$”)

**Scopus**

“protected area” OR “nature reserve” OR “nature preserve” OR “nature conserve” OR “biosphere reserve” OR “bioreserve” OR “national park” OR “wilderness area” OR “community conserved area” OR {biodiversity conservation} OR “marine reserve” OR “marine conservation area” OR “marine sanctuar*” OR “marine managed area” OR {wildlife conservation} OR “marine conserved area” OR “marine conserved territory” OR “ocean conservation area” OR “natural resource management” OR “community-based conservation” OR “managed forest” OR ({natural} AND ({monument} OR {feature})) OR ({protected} AND (“landscape” OR “seascape”)) OR ((“habitat” OR {species}) AND “management area”)
**PubMed**

“protected area*” OR “nature reserve*” OR “nature preserve” OR “nature conserve” OR “biosphere reserve” OR “bioreserve*” OR “national park” OR “wilderness area*” OR “community conserved area*” OR “biodiversity conservation” OR “marine reserve*” OR “marine conservation area*” OR “marine sanctuar*” OR “marine managed area*” OR “wildlife conservation” OR “marine conserved area*” OR “marine conserved territor*” OR “ocean conservation area*” OR “natural resource* management” OR “community-based conservation” OR “managed forest*” OR (“natural” AND (“monument” OR “feature”)) OR (“protected” AND (“landscape*” OR “seascape*”)) OR ((“habitat*" OR “species”) AND “management area*”)

**Health related strings:**

**1) General human health**

**Web of Science**

("human$" AND ("health$")) OR "self-reported health$" OR "perceived health$" OR "physical health$" OR "general health$" OR "human health$" OR "public health$" OR "self-rated health$" OR "birth weight" OR "physical activity" OR "health behavio$r" OR "population health$" OR "birth outcome$" OR (“mortality” AND ("human$" OR “people” OR "participant$" OR "infant$")) OR “morbidity" OR “birth delivery” OR “DALY$” OR “disability-adjusted life year$” OR “QALY$” OR “quality-adjusted life year$” OR “pregnancy” OR “all-cause mortality” OR “preterm birth” OR “preterm delivery” OR “health benefit$” OR “preeclampsia” OR “health promotion$” OR “preterm infant$”

**Scopus**

({human} AND “health”) OR “self-reported health” OR “perceived health” OR “physical health” OR “general health” OR “human health” OR “public health” OR “self-rated health” OR {birth weight} OR {physical activity} OR “health behavior” OR “health behaviour” OR “population health” OR “birth outcome” OR ({mortality} AND (“human” OR {people} OR “participant” OR “infant”)) OR {morbidity} OR {birth delivery} OR “DALY” OR “disability-adjusted life year” OR “QALY” OR “quality-adjusted life year” OR “pregnancy outcome” OR “all-cause mortality” OR {preterm birth} OR {preterm delivery} OR “health benefit” OR {preeclampsia} OR “health promotion” OR “preterm infant”

**PubMed**

("human*" AND ("health*")) OR "self-reported health*" OR "perceived health*" OR "physical health*" OR "general health*" OR "human health*" OR "public health*" OR "self-rated health*" OR "birth weight" OR "physical activity" OR "health behavior" OR "health behaviour" OR "population health*" OR "birth outcome*" OR (“mortality” AND ("human*" OR “people” OR "participant*" OR "infant*")) OR “morbidity" OR “birth delivery” OR “DALY*” OR “disability adjusted life year*” OR “disability-adjusted life year*” OR “QALY*” OR “quality adjusted life year*” OR “quality-adjusted life year*” OR “pregnancy outcome*” OR “all-cause mortality” OR “preterm birth” OR “preterm delivery” OR “health benefit*” OR “preeclampsia” OR “health promotion*” OR “preterm infant*”

**2) Objective well-being**

**Web of Science**

“well*being” OR “life expectanc*” OR “quality of life” OR “QOL” OR “occupational

status” OR “employment rate$” OR “work*life balance” OR “socio*economic status”

OR “income” OR “social cohesion” OR “trust” OR “human welfare” OR “literacy rate$”

OR “social capital” OR “human capital” OR “social network” OR “poverty” OR

“livelihood*” OR “economic security” OR “economic stability” OR “vulnerability”

**Scopus**

“well*being” OR “life expectanc*” OR {quality of life} OR {QOL} OR {occupational

status} OR “employment rate” OR “work*life balance” OR “socio*economic status” OR

{income} OR {social cohesion} OR {trust} OR {human welfare} OR “literacy rate” OR

{social capital} OR {human capital} OR {social network} OR {poverty} OR “livelihood”

OR “economic security” OR “economic stability” OR “vulnerability”

**PubMed**

“wellbeing” OR “well-being” OR “life expectanc*” OR “quality of life” OR “QOL” OR “occupational status” OR “employment rate*” OR “work*life balance” OR “socio*economic status” OR “income” OR “social cohesion” OR “trust” OR “human welfare” OR “literacy rate*” OR “social capital” OR “human capital” OR “social network” OR “poverty” OR “livelihood*” OR “economic security” OR “economic stability” OR “vulnerability”

**3 Subjective wellbeing (including mental health)**

**Web of Science**

(“well*being” OR “wellness” OR “health$” OR “illness*” OR “distress*” OR “disorder$” OR “symptom$” OR “feeling$” OR “disturbance$” OR “problem$”) AND (“mental” OR “psychological*” OR “emotion*” OR “mood” OR “sleep*” OR “spiritual”) OR “anxi*” OR “depress*” OR “cognitive function*” OR "attention restorat*" OR “attention deficit*” OR “mental restorat*” OR “distract*” OR “post-traumatic stress” OR “post-traumatic disorder” OR “PTSD” OR “bipolar disorder$” OR “suicide$” OR “depression$” OR “worr*” OR “fear*” OR “stress” OR “toughness” OR “loneliness” OR “social isolation$” OR “insomnia” OR “happiness” OR “sadness” OR ”apathy” OR "life satisfaction$" OR “subjective well*being” OR “subjective quality of life” OR “life meaning” OR “life purpose" OR "living condition$" OR “life condition$” OR “human flourish*” OR “academic achievement$”

**Scopus**

(“well*being” OR {wellness} OR “health” OR “illness” OR “distress” OR “disorder” OR “symptom*” OR “feeling” OR “disturbance” OR “problem”) AND ({mental} OR “psychological*” OR “emotion*” OR {mood} OR “sleep*” OR “spiritual”) OR “anxi*” OR “depress*” OR “cognitive function*” OR "attention restorat*" OR “attention deficit*” OR “mental restorat*” OR “distract*” OR “post-traumatic stress” OR “post-traumatic disorder” OR “PTSD” OR “bipolar disorder*” OR “suicide” OR “depression” OR “worr*” OR “fear*” OR {stress} OR {toughness} OR {loneliness} OR “social isolation” OR {insomnia} OR {happiness} OR {sadness} OR {apathy} OR "life satisfaction" OR {subjective quality of life} OR {life meaning} OR {life purpose} OR "living condition" OR “life condition” OR “human flourish*” OR “academic achievement”

**PubMed**

(“wellbeing” OR “well-being” OR “wellness” OR “health*” OR “illness*” OR “distress*” OR “disorder*” OR “symptom*” OR “feeling*” OR “disturbance*” OR “problem*”) AND (“mental” OR “psychological*” OR “emotion*” OR “mood” OR “sleep*” OR “spiritual”) OR “anxi*” OR “depress*” OR “cognitive function*” OR "attention restorat*" OR “attention deficit*” OR “mental restorat*” OR “distract*” OR “post-traumatic stress” OR “posttraumatic stress” OR “post-traumatic disorder” OR “posttraumatic disorder” OR “PTSD” OR “bipolar disorder*” OR “suicide*” OR “depression*” OR “worr*” OR “fear*” OR “stress” OR “toughness” OR “loneliness” OR “social isolation*” OR “insomnia” OR “happiness” OR “sadness” OR ”apathy” OR "life satisfaction*" OR “subjective quality of life” OR “life meaning” OR “life purpose" OR "living condition*" OR “life condition*” OR “human flourish*” OR “academic achievement*”

**4) Atopic and respiratory diseases**

**Web of science**

"asthma" OR "atop*" OR "eczema" OR "atopic dermatit*" OR "allerg*" OR "anaphylaxis" OR “hypersensitivity” OR “lung function” OR “respiratory disease$” OR "immunity" OR "immune response$" OR "immune regulation$" OR “anaphylactic shock” OR “inflammatory bowel disease$” OR “irritable bowel syndrome” OR “allergic disease$” OR “autoimmune disease$” OR “dermatitis” OR “inflammatory disease$” OR “ulcerative colitis” OR “immune mediated disease$” OR "asthma symptom$" OR "respiratory health$”

**Scopus**

{asthma} OR “atop*” OR {eczema} OR “atopic dermatit*” OR “allerg*” OR {anaphylaxis} OR {hypersensitivity} OR {lung function} OR “respiratory disease” OR {immunity} OR “immune response” OR “immune regulation” OR {anaphylactic shock} OR “inflammatory bowel disease” OR {irritable bowel syndrome} OR “allergic disease” OR “autoimmune disease” OR {dermatitis} OR “inflammatory disease” OR {ulcerative colitis} OR “immune mediated disease” OR “asthma symptom” OR “respiratory health”

**PubMed**

“asthma” OR “atop*” OR “eczema” OR “atopic dermatit*” OR “allerg*” OR “anaphylaxis” OR “hypersensitivity” OR “lung function” OR “respiratory disease*” OR “immunity” OR “immune response*” OR “immune regulation*” OR “anaphylactic shock” OR “inflammatory bowel disease*” OR “irritable bowel syndrome” OR “allergic disease*” OR “autoimmune disease*” OR “dermatitis” OR “inflammatory disease*” OR “ulcerative colitis” OR “immune mediated disease*” OR “asthma symptom*” OR “respiratory health*”

**5) Cancer related diseases**

**Web of Science**

"cancer$” OR “melanoma” OR “leuk$emia” OR “carcinoma” OR “neoplasm” OR “lymphoma” OR “metastasis” OR “tumo$r*” OR “lump” OR “neurofibroma” OR “malign*” OR “basal cell carcinoma” OR “squamous cell carcinoma” OR “pancreatic cancer$” OR “lung cancer$” OR “skin cancer$” OR “breast cancer$” OR “cervical cancer$” OR “ovarian cancer$” OR “prostate cancer$” OR “kidney cancer$” OR “thyroid cancer$” OR “brain cancer$” OR “colorectal cancer$” OR “gastric cancer$” OR “biliary tract cancer$” OR "oral cancer$" OR "oral carcinogenesis"

**Scopus**

“cancer” OR {melanoma} OR “leukemia” OR “leukaemia” OR {carcinoma} OR {neoplasm} OR {lymphoma} OR {metastasis} OR “tumor” OR “tumour” OR {lump} OR {neurofibroma} OR “malign*” OR {basal cell carcinoma} OR {squamous cell carcinoma} OR “pancreatic cancer” OR “lung cancer” OR “skin cancer” OR “breast cancer” OR “cervical cancer” OR “ovarian cancer” OR “prostate cancer” OR “kidney cancer” OR “thyroid cancer” OR “brain cancer” OR “colorectal cancer” OR “gastric cancer” OR “biliary tract cancer” OR "oral cancer" OR {oral carcinogenesis}

**PubMed**

“cancer*” OR “melanoma” OR “leukemia” OR “leukaemia” OR “carcinoma” OR “neoplasm” OR “lymphoma” OR “metastasis” OR “tumor*” OR “tumour*” OR “lump” OR “neurofibroma” OR “malign*” OR “basal cell carcinoma” OR “squamous cell carcinoma” OR “pancreatic cancer*” OR “ lung cancer*” OR “skin cancer*” OR “breast cancer*” OR “cervical cancer*” OR “ovarian cancer*” OR “prostate cancer*” OR “kidney cancer*” OR “thyroid cancer*” OR “brain cancer*” OR “colorectal cancer*” OR “gastric cancer*” OR “biliary tract cancer*” OR “oral cancer*” OR “oral carcinogenesis”

**6) Cardiovascular diseases**

**Web of Science**

"myocardial infarction$" OR "heart failure$" OR "acute coronary syndrome$" OR "heart disease$" OR "cardiovascular disease$" OR “hypertension$” OR “arrhythmia" OR "cardiovascular health$" OR “blood pressure” OR “coronary heart disease$” OR “cardiovascular risk factor$” OR “cardiovascular risk$” OR ((“diastolic” OR “systolic”) AND “blood pressure”) OR “cardiovascular mortality” OR “ischemic heart disease$” OR “arterial hypertension$” OR “cardiovascular disease risk$” OR “cardiovascular disease mortality” OR “dyslipid$emia” OR “chronic obstructive pulmonary disorder$” OR “COPD” OR "heart rate variability"

**Scopus**

“myocardial infarction” OR “heart failure” OR “acute coronary syndrome” OR “heart disease” OR “cardiovascular disease” OR “hypertension” OR {arrhythmia} OR “cardiovascular health” OR {blood pressure} OR “coronary heart disease” OR “cardiovascular risk factor” OR “cardiovascular risk” OR (({diastolic} OR {systolic}) AND {blood pressure}) OR {cardiovascular mortality} OR “ischemic heart disease” OR “arterial hypertension” OR “cardiovascular disease risk” OR {cardiovascular disease mortality} OR {dyslipidemia} OR {dyslipidaemia} OR “chronic obstructive pulmonary disorder” OR {COPD} OR {heart rate variability}
**PubMed**

"myocardial infarction*" OR " heart failure*" OR "acute coronary syndrome*" OR "heart disease*" OR "cardiovascular disease*" OR “hypertension*” OR “arrhythmia" OR "cardiovascular health*" OR “blood pressure” OR “coronary heart disease*” OR “cardiovascular risk factor*” OR “cardiovascular risk*” OR ((“diastolic” OR “systolic”) AND “blood pressure”) OR “cardiovascular mortality” OR “ischemic heart disease*” OR “arterial hypertension*” OR “cardiovascular disease risk*” OR “cardiovascular disease mortality” OR “dyslipidemia” OR “dyslipidaemia” OR “chronic obstructive pulmonary disorder*” OR “COPD” OR "heart rate variability"

**7) Food and nutrition security**

**Web of Science**“food secur*” OR “food insecur*” OR “food sufficien*” OR “food insufficien*” OR “food access*” OR “food availability” OR “food stability” OR “yield$ stability” OR “food utili?ation” OR “household* food secur*” OR “malnutrition” OR “an$emia” OR “undernutrition” OR "overnutrition" OR “energy intake” OR “nutri* secur*” OR “nutri* insecur*” OR “nutri* sufficien*” OR “nutri* insufficien*” OR “nutri* deficien*” OR “nutri* stability” OR “ultraproces* food” OR “proces* food” OR “junk food” OR “fast food” OR “diet composition” OR “BMI” OR “body mass index” OR “stunting” OR “obes*” OR "diabetes" OR “dietary sustainability” OR (“diet” AND ("human$" OR “people” OR "participant$")) OR “dietary diversity” OR “food supply diversity” OR “dietary restriction$” OR “food preference$” OR “food taboo$”

**Scopus**

“food secur*” OR “food insecur*” OR “food sufficien*” OR “food insufficien*” OR “food access” OR {food availability} OR {food stability} OR “yield stability” OR “food utili?ation” OR “household* food secur*” OR {malnutrition} OR {anemia} OR {anaemia} OR {undernutrition} OR {overnutrition} OR {energy intake} OR “nutri* secur*” OR “nutri* insecur*” OR “nutri* sufficien*” OR “nutri* insufficien*” OR “nutri* deficien*” OR “nutri* stability” OR “ultraproces* food” OR “proces* food” OR {junk food} OR {fast food} OR {diet composition} OR {BMI} OR {body mass index} OR {stunting} OR “obes*” OR {diabetes} OR “dietary sustainability” OR (“diet” AND ("human" OR “people” OR "participant")) OR “dietary diversity” OR “food supply diversity” OR “dietary restriction” OR “food preference” OR “food taboo”

**PubMed**

“food secur*” OR “food insecur*” OR “food sufficien*” OR “food insufficien*” OR “food access*” OR “food availability” OR “food stability” OR “yield* stability” OR “food utilisation” OR “food utilization” OR “household* food secur*” OR “malnutrition” OR “undernutrition” OR "overnutrition" OR “energy intake” OR “nutritional secur*” OR “nutritional insecur*” OR “nutritional sufficien*” OR “nutritional insufficien*” OR “nutritional deficien*” OR “nutritional stability” OR

“nutrition secur*” OR “nutrition insecur*” OR “nutrition sufficien*” OR “nutrition insufficien*” OR “nutrition deficien*” OR “nutrition stability” OR “ultraprocessed food” OR “processed food” OR “junk food” OR “fast food” OR “diet composition” OR “BMI” OR “body mass index” OR “stunting” OR “obes*” OR "diabetes" OR “dietary sustainability” OR (“diet” AND ("human" OR “people” OR "participant")) OR “dietary diversity” OR “food supply diversity” OR “dietary restriction*” OR “food preference*” OR “food taboo*”

**8) Infectious diseases**

**Web of Science**

"infectious disease$" OR "communicable disease$" OR “zoonos*” OR “emerging disease$” OR “emerging infecti*” OR “vector-borne disease$” OR “water-borne disease$” OR “bacterial disease$” OR “viral infection” OR “viral disease$” OR “fungal infection” OR “fungal disease$” OR “bacterial infection” OR “transmitted disease$” OR “sexual* transmitted infection” OR “dilution effect$” OR “disease$ emergence” OR “diversity-disease$” OR “disease outbreak$” OR “zoonotic disease$” OR “disease transmission$” OR “infectious-disease$ risk”

**Scopus**

“infectious disease” OR “communicable disease” OR “zoonos*” OR “emerging disease” OR “emerging infecti*” OR “vector-borne disease” OR “water-borne disease” OR “bacterial disease” OR {viral infection} OR “viral disease” OR {fungal infection} OR “fungal disease” OR {bacterial infection} OR “transmitted disease” OR “sexual* transmitted infection” OR “dilution effect” OR “disease emergence” OR “diversity-disease*” OR “disease outbreak’” OR “zoonotic disease*” OR “disease transmission” OR “infectious-disease risk”

**PubMed**

“infectious disease*” OR “communicable disease*” OR “zoonos*” OR “emerging disease*” OR “emerging infecti*” OR “vector-borne disease*” OR “water-borne disease*” OR “bacterial disease*” OR “viral infection” OR “viral disease*” OR “fungal infection” OR “fungal disease*” OR “bacterial infection” OR “transmitted disease*” OR “sexual transmitted infection” OR “sexually transmitted infection” OR “dilution effect*” OR “disease emergence” OR “diseases emergence” OR “diversity-disease*” OR “disease outbreak*’” OR “zoonotic disease*” OR “disease transmission*” OR “infectious-disease* risk”

**9) Other Non-communicable diseases**

**Web of Science**

"non*communicable disease$" OR "Alzheimer’s disease" OR "arthritis" OR "Crohn’s disease$" OR “crohn disease$” OR "liver disease" OR "fibromyalgia" OR "Parkinson’s disease" OR "Parkinson disease" OR "dementia" OR "neurodegenerative disease$" OR “cognitive function” OR “Kufor-Rakeb syndrome” OR “rheumatoid arthritis” OR “cognitive impairment” OR “neurodegenerative disorder$” OR “acute ischemic stroke” OR “Parkinson disease” OR “kidney disease” OR “cerebrovascular disease” OR “muscular dystrophy” OR “chronic fatigue syndrome” OR “sleep disorder$” OR “sickle cell an$emia” OR “osteoarthritis” OR “epilepsy” OR “osteoporosis” OR “substance abuse” OR “stroke” OR "multiple sclerosis"

**Scopus**

“non*communicable disease*” OR {Alzheimer’s disease} OR {arthritis} OR “Crohn’s disease” OR “crohn disease” OR {liver disease} OR {fibromyalgia} OR {Parkinson’s disease} OR {Parkinson disease} OR {dementia} OR “neurodegenerative disease” OR {cognitive function} OR “Kufor-Rakeb syndrome” OR {rheumatoid arthritis} OR {cognitive impairment} OR “neurodegenerative disorder” OR {acute ischemic stroke} OR {Parkinson disease} OR {kidney disease} OR {cerebrovascular disease} OR {muscular dystrophy} OR {chronic fatigue syndrome} OR “sleep disorder” OR {sickle cell anemia} OR {sickle cell anaemia} OR {osteoarthritis} OR {epilepsy} OR {osteoporosis} OR {substance abuse} OR {stroke} OR {multiple sclerosis}

**PubMed**

"noncommunicable disease*" OR "non-communicable disease*" OR "Alzheimer’s disease" OR "arthritis" OR "Crohn’s disease" OR “crohn disease*” OR "liver disease" OR "fibromyalgia" OR "Parkinson’s disease" OR "Parkinson disease" OR "dementia" OR "neurodegenerative disease*" OR “cognitive function” OR “Kufor-Rakeb syndrome” OR “rheumatoid arthritis” OR “cognitive impairment” OR “neurodegenerative disorder*” OR “acute ischemic stroke” OR “Parkinson disease” OR “kidney disease” OR “cerebrovascular disease” OR “muscular dystrophy” OR “chronic fatigue syndrome” OR “sleep disorder*” OR {sickle cell anemia} OR {sickle cell anaemia} OR “osteoarthritis” OR “epilepsy” OR “osteoporosis” OR “substance abuse” OR “stroke” OR "multiple sclerosis"

**Study design**

**Reviews**

**Web of Science**

((“systematic” OR “meta-analysis”) AND (“review$”)) OR "meta-analysis" OR "meta-analytic" OR “evidence synthesis” OR “review$” OR “narrative review$” OR “literature review$” OR “systematic” OR “scoping review$” OR “rapid review$” OR “mapping review$” OR “meta-synthesis” OR “mixed method review$” OR “integrative review$” OR “umbrella review$” OR “knowledge synthesis”

**Scopus**

(({systematic} OR “meta-analysis”) AND (“review”)) OR “meta-analysis” OR “meta-analytic” OR {evidence synthesis} OR “review” OR “narrative review” OR “literature review” OR {systematic} OR “scoping review” OR “rapid review” OR “mapping review” OR “meta-synthesis” OR “mixed method review” OR ”integrative review” OR “umbrella review” OR {knowledge synthesis}

**PubMed**

((“systematic” OR “meta-analysis”) AND (“review*”)) OR "meta-analysis" OR "meta-analytic" OR “evidence synthesis” OR “review*” OR “narrative review*” OR “literature review*” OR “systematic” OR “scoping review*” OR “rapid review*” OR “mapping review*” OR “meta-synthesis” OR “mixed method review*” OR “integrative review*” OR “umbrella review*” OR “knowledge synthesis”

**Primary studies**

**Web of Science**

“cross-sectional” OR “cohort” OR “retrospective” OR “prospective” OR “quantitative” OR “longitudinal” OR “case-control” OR “case-cohort” OR “cohort study” OR “case-crossover” OR “logistic regression$” OR “regression$” OR “association$” OR “relationship$” OR "survey" OR "multicase-control" OR "metagenome analysis" OR "statistical analysis" OR "empirical evidence" OR "empirical" OR "correlation$" OR "questionnaire" OR "quasi-experiment" OR "evidence-based" OR “intervent*” OR "cross-over" OR "comparison" OR " molecular-based" OR "associated" OR "multi-disciplinary” OR "controlled trial$" OR "preliminary study"

**Scopus**

“cross-sectional” OR {cohort} OR {retrospective} OR {prospective} OR {quantitative} OR {longitudinal} OR “case-control” OR “case-cohort” OR {cohort study} OR “case-crossover” OR “logistic regression” OR “regression” OR “association” OR “relationship” OR {survey} OR “multicase-control” OR {metagenome analysis} OR {statistical analysis} OR {empirical evidence} OR {empirical} OR ”correlation” OR {questionnaire} OR “quasi-experiment” OR “evidence-based” OR “intervent*” OR {cross-over} OR {comparison} OR “molecular-based” OR {associated} OR “multi-disciplinary” OR “controlled trial” OR “preliminary study”

**PubMed**

“cross-sectional” OR “cohort” OR “retrospective” OR “prospective” OR “quantitative” OR “longitudinal” OR “case-control” OR “case-cohort” OR “cohort study” OR “case-crossover” OR “logistic regression*” OR “regression*” OR “association*” OR “relationship*” OR "survey" OR "multicase-control" OR "metagenome analysis" OR "statistical analysis" OR "empirical evidence" OR "empirical" OR "correlation*" OR "questionnaire" OR "quasi-experiment" OR "evidence-based" OR “intervent*” OR "cross-over" OR "comparison" OR

"molecular-based" OR "associated" OR "multi-disciplinary” OR "controlled trial*" OR "preliminary study"

**Participants (To exclude the animal studies)**

**Web of Science**

NOT "mice" OR "mouse" OR "rodent*" OR "non-human" OR “rat" OR “rats” OR (“animal*” AND “model*”)

**Scopus**

AND NOT {mice} OR {mouse} OR “rodent*” OR “non-human” OR “rat” OR (“animal*” AND “model*”)

**PubMed**

NOT (animals[MeSH Terms] NOT humans[MeSH Terms])
