# Supplementary material for: What evidence exists regarding the impact of biodiversity on human health and well-being? A systematic map protocol
Source: Environ Evid. 2024 Apr 27;13:11. doi: 10.1186/s13750-024-00335-4 (PMC11378774; doi:10.1186/s13750-024-00335-4)
Supplement: Supplementary file 5 — Additional file 5: Data coding system. [file 13750_2024_335_MOESM5_ESM.docx]

**Supplementary file 5: Data coding system**

**Section 1 Study characteristics** (from title, abstract and method parts of included studies)

- 1. **Study ID**

**1.2 Person of extraction**

- HL
- RJ
- ETC
  1. **Screen date**

**1.3 Publication year**

**1.4 Authors**

**1.5 Year**

**1.6 Title**

**1.7 Abstract**

**1.8 Study design**

1.8.1 Secondary studies: (based on how the authors define their study design, we will not reclassify the studies)

- Narrative review
- Systematic review only
- Systematic map
- Systematic review and meta-analysis

1.8.2 Primary study (studies based on the data which is obtained first-hand)

- Experimental study
- Cross-sectional study
- Cohort
- Longitudinal study

**Section 2 Population** (from title, abstract and method parts)

**2.1 Scales of population**

- Individual
- Household
- Community
- Country
- Intercontinental
- Global

**2.2 Spatial extent**

- Log-10 scale km^2: 1-8 (10^0^ - 10^8^ Area of the planet)
- NA

**2.3 Spatial unit** (referring to the NUTS system)

- Administrative region (e.g. Skåne)
- Country
- Continent
- Global

**2.4 Age range**

- All ages
- Children (less than 15 years old)
- Youth (15-24 years old)
- Adults (24-60 years old)
- Elderly people (older than 60 years old)

**2.5 Sex**

- Male
- Female

**2.6 Area**

- Urban/rich
- Peri-urban
- Rural/poor

**Section 3 Exposure** (From title, abstract and method parts)

**3.1 Which biodiversity forms?** (Clearly claimed or indicated with the searching strings used in secondary studies, check multiple if the study includes more than one forms)

- Blue space only
- Food production only
- Green space only
- Microbiome only
- Protected areas only
- General biodiversity (we will inductively code forms of biodiversity under this category)

**3.2 Biodiversity level** (referring to CBD definition and Millennium)

- Genetic
- Species
- Community
- Ecosystem

**3.3 Biodiversity aspect** (referring to CBD definition and Millennium)

- Taxonomic
- Phylogenetic
- Functional

**Section 4 Comparators** (from methods part)

**4.1 Yes-No** (e.g., a study population that has been exposed to natural biodiversity vs. one that hasn’t been exposed)

**4.2 Level of biodiversity** (e.g., any comparison between settings with lower and higher amounts of biodiversity exposure)

**4.3 Time**

**4.4 Distance**

**4.5 External vs. subjective measurement**

- Externally measured biodiversity (biodiversity present at a location, could be assessed with ecological field techniques, comprehensive monitoring or counting)
- Subjectively measured (perceived) biodiversity (biodiversity people think is at a location, could be measured by survey, questionnaires, observational research)

**Section 5 Outcomes** (from title, abstract or method part)

**5.1 Which human health component?**

- Atopic and respiratory disease only
- Cancer related disease only
- Cardiovascular and respiratory disease only
- Food and nutrition security only
- Mental health and subjective wellbeing only
- Objective wellbeing only
- Mental health only
- Infectious disease only
- Non-communicable disease only
- General human health (we will specify the sub-groups at second level)

**5.2 Assessment of human health and wellbeing?**

- Self-reported
- External measurement
- NA
